# The association of semaphorin 5A with lymph node metastasis and adverse prognosis in cervical cancer

**DOI:** 10.1186/s12935-018-0584-1

**Published:** 2018-06-22

**Authors:** Jian-Bing Xiao, Xin-Lei Li, Le Liu, Geng Wang, Song-Nan Hao, Hui-Juan Dong, Xue-Min Wang, Ya-Fang Zhang, Hui-Dong Liu

**Affiliations:** 1 Department of Anatomy, Harbin Medical University, 194 Xuefu Road, Harbin, 150081 China; 2 Department of Orthopedics, Harbin Fifth Hospital, 27 Jiankang Road, Harbin, 150040 China; 3 Department of CT Scan, Heilongjiang Province Red Cross Hospital, 32 Hexing Road, Harbin, 150008 China; 4 Department of Anesthesiology, Heilongjiang Province Red Cross Hospital, 32 Hexing Road, Harbin, 150008 China

**Keywords:** Cervical cancer, Lymph node metastasis, Lymphangiogenesis, Prognosis, Semaphorin 5A

## Abstract

**Background:**

Semaphorin 5A has been linked to tumor growth, invasion, and metastasis in pancreatic cancer. However, the role of semaphorin 5A in cervical cancer is not known. Our aim is to investigate the prognostic value of semaphorin 5A and its potential role in lymphangiogenesis and invasion in cervical cancer.

**Methods:**

In this study, pathological features and clinical data of 232 cervical cancer patients were retrospectively reviewed. Semaphorin 5A protein and mRNA expression was detected by immunohistochemistry and quantitative real-time reverse transcription-polymerase chain reaction, respectively. In vitro, we determined the role and mechanistic pathways of semaphorin 5A in tumor progression in cervical carcinoma cell lines.

**Results:**

Semaphorin 5A expression was significantly higher in stage IIb tumors than in stage Ia, Ib, and IIa tumors. High semaphorin 5A expression was significantly associated with pelvic lymph node metastasis, lymphovascular permeation, and poor survival. Semaphorin 5A induced lymphangiogenesis through a plexin-B/Met/vascular endothelial growth factor-C pathway. Semaphorin 5A also increased cervical cancer cell invasion by stimulating the expression and activity of matrix metalloproteinase-2 and matrix metalloproteinase-9 via PI3K/AKT and plexin-B3.

**Conclusion:**

Our findings indicate that semaphorin 5A may represent a poor prognostic biomarker and anti-metastasis therapeutic target in cervical cancer.

**Electronic supplementary material:**

The online version of this article (10.1186/s12935-018-0584-1) contains supplementary material, which is available to authorized users.

## Background

Cervical cancer remains the leading malignancy among women in China, with an estimated incidence of 132,000 new cases and mortality of 30,000 in 2011 [1]. Lymph node metastasis (LNM) is an important factor for tumor recurrence and disease progression. The prognosis for patients with lymph node metastases is inversely correlated to the number of nodes involved. Bilateral lymph node involvement confers a much poorer prognosis than unilateral involvement. Patients with lymph node metastases show a markedly lower 5-year survival rate (41.1%) than those without such metastases (91.9%). Tumor recurrence is significantly increased in patients with lymph node metastases. Seven of 94 patients (7.4%) have lymph node metastases. Five patients develop recurrent cancer [2, 3]. A better understanding of the underlying molecular mechanisms of LNM is required to identify prognostic markers and therapeutic targets that will help to prevent LNM.

Semaphorins, a large family of secreted glycosylphosphatidylinositol-linked transmembrane proteins, is first identified for their central role in axonal guidance and nervous system development [4, 5]. Recent studies have suggested that the semaphorin family is widely distributed in many tissues and organs apart from the nervous system and is involved in cell migration, blood vessel growth, tumor progression, and metastasis [6, 7]. The semaphorin V subfamily has been found to promote angiogenesis by increasing endothelial cell proliferation and migration and decreasing apoptosis [8]. However, the role of semaphorin V subfamily members in lymphangiogenesis is unknown. A study demonstrated that the expression of SEMA5A, a member of the semaphorin V subfamily, is associated with tumor growth, invasion, and metastasis in pancreatic cancer cells [9]. However, SEMA5A expression and its correlation with lymphangiogenesis, LNM, and clinicopathological parameters in cervical cancer have not been reported. Thus, we evaluated SEMA5A expression and its association with lymphangiogenesis, LNM, histopathological characteristics, and survival patterns in patients with cervical cancer. We also investigated the role and molecular mechanism of action of SEMA5A in lymphangiogenesis and invasion.

## Materials and methods

### Patients and tissue samples

Tissue specimens were obtained from the Tumor Hospital of Harbin Medical University and First Clinical College of Harbin Medical University. Two hundred and thirty-two cervical cancer patients who underwent cervical surgery and dissection of pelvic/aortic lymph nodes between July 2004 and December 2011 were included in the study. Pathological features and clinical data of patients were obtained retrospectively from the hospital database. The median follow-up period was 96 months (range 12–97 months). None of the patients had any other specified carcinomas within 5 years of their cervical cancer diagnosis. The median age at the time of surgery was 49 years (range 23–69 years). Surgical treatments included cone biopsy in 27 patients (11.6%) and radical hysterectomy in 205 patients (83.4%). After surgery, 215 patients were treated with adjuvant therapy. Seventeen patients (7.3%) did not receive adjuvant therapy because they were at minimal risk or for other reasons.

Tumor stage, tumor grade, and LNM presence were assessed using HE-stained sections. Of the 232 patients, 156 patients (67.2%) had squamous cell carcinoma, 61 patients (26.3%) had adenocarcinoma, and 15 patients (6.5%) had adenosquamous carcinoma. Lymph node involvement was present in 121 of the 232 patients. Low, intermediate, and high tumor grades were confirmed in 47, 104, and 81 patients, respectively. Furthermore, 12 cases were stage Ia, 79 cases were stage Ib, 87 cases were stage IIa, and 54 cases were stage IIb according to the International Federation of Gynecology and Obstetrics (FIGO) staging system. The mean tumor size was 2.75 cm, and the mean depth of invasion was 10.3 mm. The study procedures were in accordance with the guidelines of the National Research Council and approved by the Research Ethics Committee of Harbin Medical University.

### Cell lines

The HeLa human cervical carcinoma cell line, Siha and Caski human papillomavirus 16-positive cervical carcinoma cell lines were used as in vitro cervical cancer models. The HeLa cell line was provided from the Cancer Institute of Harbin Medical University. Another two cervical carcinoma cell lines Siha and Caski were obtained from the American Type Culture Collection (ATCC, Manassas, USA). HeLa cell line was cultured in RPMI 1640 (Invitrogen, Carlsbad, CA, USA). The Siha and Caski cell lines were cultured in DMEM (Invitrogen) supplemented with 10% FBS in a 5% CO_2_ at 37 °C.

### Immunohistochemistry for SEMA5A and lymphatic vessel endothelial hyaluronan receptor-1

Immunohistochemistry was used to evaluate SEMA5A and lymphatic vessel endothelial hyaluronan receptor-1 (LYVE-1) expression in cervical cancer tissues. For immunostaining, 4-μm-thick tissue sections were deparaffinized in xylene, rehydrated through graded alcohol, and treated with 5% H_2_O_2_ to quench endogenous peroxidase activity. After antigen retrieval in citrate buffer, the sections were incubated with polyclonal goat anti-human SEMA5A (1:400 dilution; sc-67953, Santa Cruz Biotechnology, CA, USA) and polyclonal rabbit anti-human LYVE-1 primary antibodies (1:200 dilution; sc-19316, Santa Cruz Biotechnology) at 4 °C overnight. Species-appropriate biotinylated secondary antibodies were used for antigen detection. All subsequent immunohistochemistry procedures were carried out as previously described [10]. The negative control was obtained by using PBS instead of the primary antibody (Additional file 1: Figure S1A). Specimens with known SEMA5A expression served as positive controls (Additional file 1: Figure S1B). Scoring of SEMA5A was determined according to Fanourakis et al. [11]. Immunoreactivity for SEMA5A was evaluated according to the extent and intensity of staining. For statistical analysis, we divided patients into two groups. Tumor tissue with a score of ≥ 5 for SEMA5A staining was defined as the high expression and tumor tissue with a score of < 5 was regarded as the low expression. To assess lymphangiogenesis, lymphatic microvessel density (LMVD) was determined by LYVE-1 immunostaining as previously reported [12]. The area containing the most LYVE-1-stained vessels (hot spot) was identified by scanning the sections at low magnification. LMVD was then measured by counting the number of LYVE-1-positive vessels from three areas of the highest vessel density/section at 200×. Each brown-stained lumen was regarded as a single countable microvessel. Three sections/tumors were analyzed. The analysis of SEMA5A and LYVE-1 immunostaining was conducted by two independent observers without knowledge of any other variables or clinical data. Cases of disagreement were reanalyzed until a consensus was reached.

### Quantitative real-time reverse transcription-polymerase chain reaction

Quantitative real-time reverse transcription-polymerase chain reaction (RT-PCR) analysis was performed using an ABI PRISM 7000 Sequence Detection System (Applied Biosystems/Life Technologies, Foster, CA, USA). The following primer sequences were used: SEMA5A, 5′-GATCTATGGCATCTTTACCACCAA-3′ and 5′-TGG CGCTCAGGTTGAAGAC-3′; VEGF-C, 5′-AAGGAGGCTGGCAACATA-3′ and 5′-TGGCAGGGAACGTCTAAT-3′; matrix metalloproteinase (MMP)-2, 5′- CAGGAGGAGAAGGCTGTGTT-3′ and 5′-AGGGTGCTGGCTGAGTAGAT-3′; MMP-9, 5′-AGAACCAATCTCACCGACAGG-3′ and 5′-CGACTCTCCACGCATCTCT-3′; and GAPDH, 5′-GAGTCAACGGATTTGGTCGTA-3′ and 5′-ATGGGATTTCCATTGATGACA-3′. *SEMA5A*, *MMP*-*2*, *MMP*-*9*, and *GAPDH* expression levels were assessed using a fluorescence-based real-time detection method. For quantitative analyses, *SEMA5A*, *MMP*-*2*, and *MMP*-*9* mRNA expression were normalized to *GAPDH* mRNA expression using previously published protocols [10].

### Immunofluorescent antibody staining and ELISA for vascular endothelial cell growth factor-C

For immunofluorescent antibody staining, chamber slides were precoated with SEMA5A (100 ng/mL; R&D Systems, Minneapolis, MN, USA) overnight at 4 °C. HeLa human cervical cancer cells were seeded into SEMA5A-coated and uncoated chamber slides for 24 h. The slides were fixed with fresh 4% paraformaldehyde for 15 min at room temperature, blocked in PBS containing 10% normal serum, and incubated with vascular endothelial growth factor (VEGF)-C primary antibody (1:100 dilution; sc-9047, Santa Cruz Biotechnology) for 2 h. The slides were incubated with fluorescein isothiocyanate (FITC)-conjugated IgG (1:100 dilution; Sigma-Aldrich, Beijing, China) for 1 h, counterstained with the fluorescent nuclear stain PI (Sigma-Aldrich, Beijing, China) for 5 min, and examined under a Nikon fluorescence microscopy.

VEGF-C protein levels in cell culture supernatants were determined to us the VEGF-C ELISA Kit (R&D Systems), according to the manufacturer’s instructions.

### Western blot

Western blot was carried out as previously described [10]. The following antibodies were used for western blot: anti-SEMA5A (1:200 dilution), anti-VEGF-C (1:200 dilution), anti-Tubulin (1:500 dilution; MAB1637, Chemicon International), anti-phosphotyrosine (1:100 dilution; clone 4G10, 16-105, Upstate Biotech), anti-Met (1:100 dilution; SP260, sc-162, Santa Cruz Biotechnology), anti-phosphorylated AKT (1:500 dilution; #9271, Cell Signaling Technology, Danvers, MA), anti-MMP-2 (1:300 dilution; sc-53630, Santa Cruz Biotechnology), and anti-MMP-9 (1:300 dilution; sc-6840, Santa Cruz Biotechnology).

### Met and phosphoinositide 3-kinase inhibition

To block the kinase activity of Met, the competitive Met inhibitor SU11274 (Sigma-Aldrich, St. Louis, MO, USA) and a Met neutralizing antibody (R&D Systems) were used. Cells were treated with 100 ng/mL SEMA5A in the presence or absence of SU11274 (2.5 μM) or Met neutralizing antibody (2 μg/mL) for 24 h. The concentration of SU11274 was used at 2.5 μM because inhibitory effect on cell growth was apparent with over 2.5 µM of SU11274 [13–15]. To block phosphoinositide 3-kinase (PI3K)/AKT signaling, the PI3K inhibitor LY294002 (Cell Signaling Technology, Danvers, MA) was used. Cells were treated with 50 μmol/L LY294002 for 24 h. Cells were treated with 50 μmol/L LY294002 for 24 h. The concentration of LY294002 was set at 50 μmol/L in accordance with previous study [16].

### HGF cell treatment, Met neutralizing antibody and siRNA transfections

Recombinant hepatocyte growth factor (HGF) was purchased from CalBiochem (San Diego, CA). The human cervical carcinoma cells were treated with HGF (30 ng/mL) and then incubated in a humidified incubator at 37 °C for 24 h. To examine the downstream signaling pathways involved in HGF treatment, cells were pretreated with the Met neutralizing antibody (2 μg/mL) or transfected with Met siRNA for 24 before addition of HGF (30 ng/mL). The small interfering RNAs (siRNAs) against Met and control siRNA were purchased from Santa Cruz Biotechnology (Santa Cruz, CA). VEGF-C in the medium was assayed using by RT-PCR and ELISA.

### *Plexin*-*B3* knockdown

*Plexin*-*B3* expression was knocked down to use short hairpin RNA (shRNA) interference technology. HeLa cells were transfected with plexin-B3-specific shRNA (5′-AGCAGATGGTGGAGAGGTA-3′) or a scrambled shRNA control (5′-GGCTACGTCCAGGAGCGCA-3′) using Lipofectamine (Invitrogen, Carlsbad, CA, USA). *Plexin*-*B3* knockdown was confirmed by RT-PCR analysis.

### Invasion assays

Invasion assays were performed as described previously [9, 17]. Briefly, HeLa, Siha, and Caski cells were treated with 100 ng/mL recombinant human SEMA5A in the presence or absence of 1 μM GM6001, a MMP inhibitor (Calbiochem, La Jolla, CA, USA). After 5 h, cells (1 × 10^5^) were seeded onto 6.5-mm Costar transwells coated with matrigel (Corning, Cambridge, MA). After incubation for 24 h at 37 °C, cells from the top of the transwell chambers were removed to use a cotton swab, and the percentage of invaded cells was calculated.

### Sema5A cDNA transfection

HeLa cells were transfected with a mammalian expression vector containing full-length mouse Sema5A tagged with the FLAG epitope (HeLa-Sema5A) or empty vector alone (HeLa-control) as described previously [8].

### Gelatin zymography

Gelatinolytic activity was determined to use zymography as previously described [18]. Briefly, culture supernatants were collected and centrifuged. The supernatants were added to sodium dodecyl sulfate (SDS) sample buffer without mercaptoethanol and electrophoresed on an 8% polyacrylamide gel containing 1.5 mg/mL gelatin. The gel was washed with 2.5% Triton X-100 to remove SDS and incubated with developing buffer (50 mmol/L Tris–HCl, 0.2 mol/L NaCl, 5 mmol/L CaCl_2_, and 0.02% Brij-35) overnight at 37 °C. Gelatinolytic bands were visualized by staining with Coomassie blue R-250 and destaining with Coomassie blue R-250 destaining solution until all lytic bands became clear. The gelatinolytic bands were quantified to use Image Acquisition and Analysis Systems (Ultra-Violet Products, Biospectrum HR410, USA).

### Statistical analysis

Data were analyzed to use SPSS for Windows version 17.0 (Chicago, IL, USA). Disease-free survival (DFS) was defined as the time interval between the end of primary therapy and the first evidence of disease progression. Overall survival (OS) was defined as the time interval from the date of surgery until the date of cervical cancer death. Univariate analysis of DFS and OS was carried out using Kaplan–Meier plots, and statistical significance between survival curves was assessed using the log-rank test. To assess the independent value of different pretreatment variables on survival in the presence of other variables, multivariate analysis was carried out using the Cox proportional hazards model. Only significant variables in the univariate analysis were used in the Cox regression analysis. Probability for stepwise entry and removal was set at 0.05 and 0.10, respectively. Chi square and Fisher’s exact tests were used to examine the association between SEMA5A expression and clinicopathological parameters. A *P* value < 0.05 was considered significant.

## Results

### SEMA5A mRNA and protein expression according to tumor stage

**Fig. 1:**
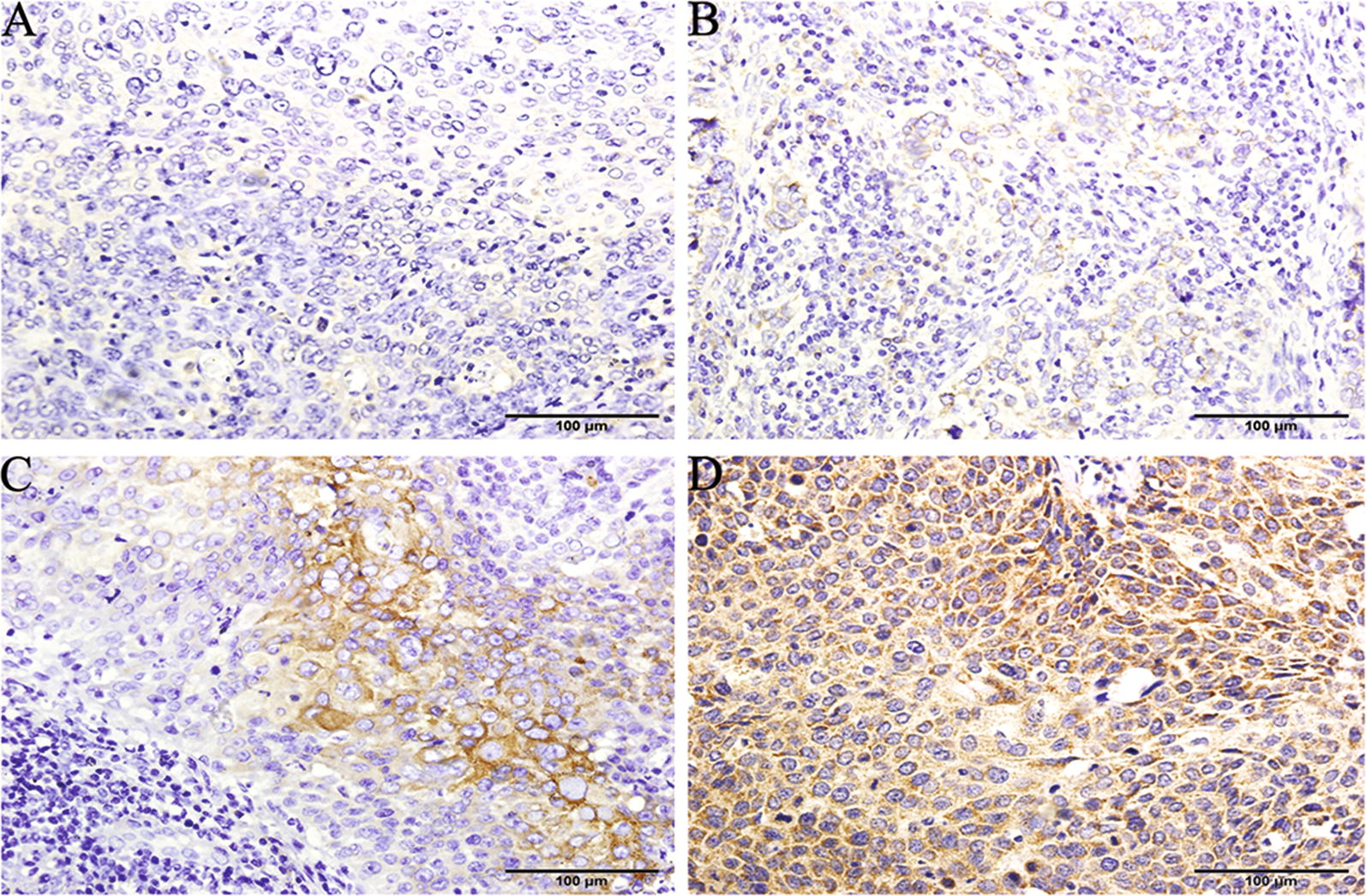
SEMA5A protein expression in human cervical cancer tissues. SEMA5A immunohistochemical staining was performed on tumor tissues from patients with different stages of cervical cancer. Almost negative or weak staining intensity was observed in **A** stage Ia, **B** Ib, and **C** IIa tumor tissues, whereas strong intensity of cytoplasmic staining was observed in 80–90% of cervical cancer cells from **D** stage IIb tumor tissues. **A**–**D** ×200 magnification. *SEMA5A* semaphorin 5A

The constitutive expression of SEMA5A protein differed according to cervical cancer stage. Of the 232 cervical cancer patients, 123 patients had high SEMA5A expression. SEMA5A protein expression was significantly higher in tumor tissues from stage IIb patients than in tumors tissues from stage Ia, Ib, and IIa patients (*P *< 0.05). Immunostaining demonstrated that SEMA5A was highly expressed in cervical cancer cells from stage IIb tumor tissues, comparable to that seen in nonsmall cell lung carcinoma (NSCLC), which was used as the positive control (Additional file 1: Figure S1B), but was expressed only at very low levels in stage Ia, Ib, and IIa tumor tissues (Fig. 1). *SEMA5A* mRNA level was also significantly higher in stage IIb tumors than in stage Ia, Ib, and IIa tumors (*P *< 0.05) but was not significantly different among stage Ia, Ib, and IIa tumors (Table 1). Thus, the RT-PCR analysis shows that the data of *SEMA5A* mRNA is increased in stage IIb tumors and supports *SEMA5A* protein expression demonstrated by IHC analysis.

**Table 1:** *SEMA5A* mRNA expression according to cervical cancer stage

FIGO stage	Lymph node metastasis	n	*SEMA5A* expression^a^
Ia	No	11	1.03 ± 0.15^b^
Ib	No	20	1.82 ± 0.24^b^
IIa	No	31	2.14 ± 0.18^b^
IIb	Yes	24	3.78 ± 0.23

*SEMA5A* mRNA expression in cervical cancer tissue (n = 86) was examined using quantitative real-time reverse transcription-polymerase chain reaction. *SEMA5A* mRNA expression was compared between stage IIb tumors and stage Ia, Ib, and IIa tumors

*FIGO* Federation of Gynecology and Obstetrics, *SEMA5A* semaphorin 5A

^a^Data are expressed as the mean ± standard deviation of at least three independent experiments

^b^*P* < 0.05 versus stage IIb

### Relationship of SEMA5A expression to LNM and lymphangiogenesis

**Fig. 2:**
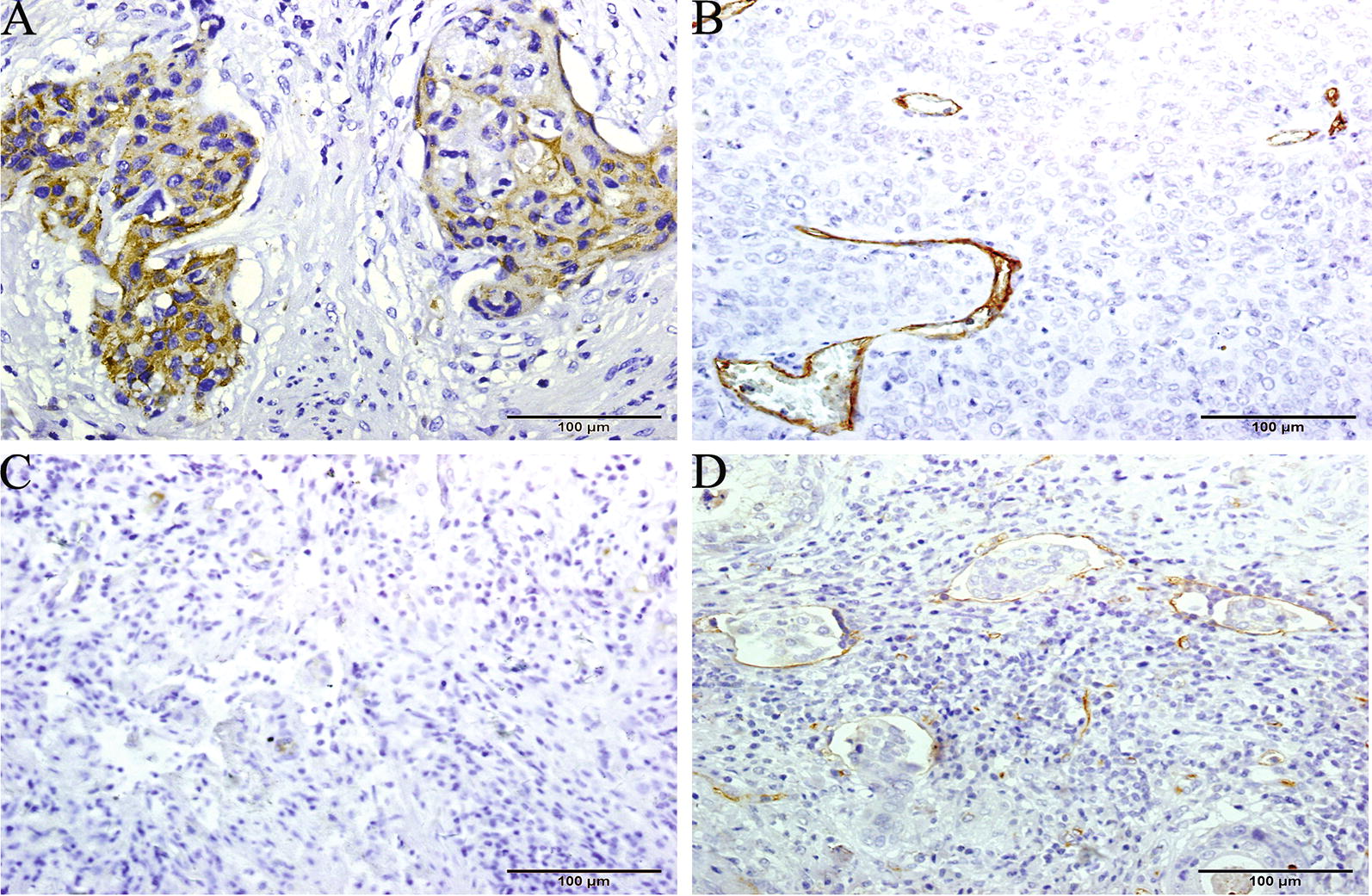
Expression patterns of SEMA5A and LYVE-1 in metastatic and nonmetastatic cervical cancers. **A** SEMA5A protein was present in cervical cancer cells that metastasized to the regional lymph nodes. Immunohistochemical staining of LYVE-1 in **B** metastatic and **C** nonmetastatic cervical cancers. Numerous peritumoral LYVE-1-stained lymphatics were observed in metastatic cervical cancer specimens, whereas few peritumoral LYVE-1-stained lymphatics were observed in nonmetastatic cervical cancer specimens. **D** The typical lymphovascular invasions with tumor cell clusters in a LYVE-1-stained lymphatic vessels. **A** ×200 magnification **B**–**D** ×100 magnification. *LYVE* lymphatic vessel endothelial hyaluronan receptor-1, *SEMA5A* semaphorin 5A

The relationship of SEMA5A expression to LNM and lymphangiogenesis is demonstrated in Table 2. High SEMA5A expression was detected at a significantly greater frequency in metastatic patients than in nonmetastatic patients (*P* < 0.001). In addition, tumor cells that metastasized to the pelvic/aortic lymph nodes also showed high SEMA5A protein expression (Fig. 2A). Peritumoral LYVE-1-positive vessels were commonly seen in patients with LNM (Fig. 2B) and infrequently detected in patients without LNM (Fig. 2C). LYVE-1-positive LMVD was significantly higher in patients with LNM compared with those without LNM (9.1 ± 0.5 microvessels/field vs. 6.7 ± 0.8 microvessels/field; *P *< 0.001). LYVE-1-positive lymphatic vessels invaded by tumor clusters were occasionally observed (Fig. 2D). Lymphovascular invasion was detected in 42 cervical cancer cases. The tumor cells within these lymphatic vessels were more often judged to have high SEMA5A expression. Among 42 lymphovascular invasions, high SEMA5A expression was evident in 33 (78.5%), in 30 with LNM and in 3 without LNM. SEMA5A high expression patients were more likely than low expression patients to have lymph node metastasis (P < 0.001).

**Table 2:** SEMA5A expression, LMVD and lymphovascular invasion in metastatic group vs. nonmetastatic group

	Metastatic group	Nonmetastatic group	*P*
n	121	111	
SEMA5A high expression	66.7%	43.3%	< 0.001
LMVD	9.1 ± 0.5^a^	6.7 ± 0.8^a^	< 0.001
Lymphovascular invasion	30	3	< 0.001

*LMVD* lymphatic microvessel density microvessels/field

^a^Microvessels/field are expressed as the mean ± standard deviation of at least three independent experiments

### Association of SEMA5A expression with DFS and OS

**Fig. 3:**
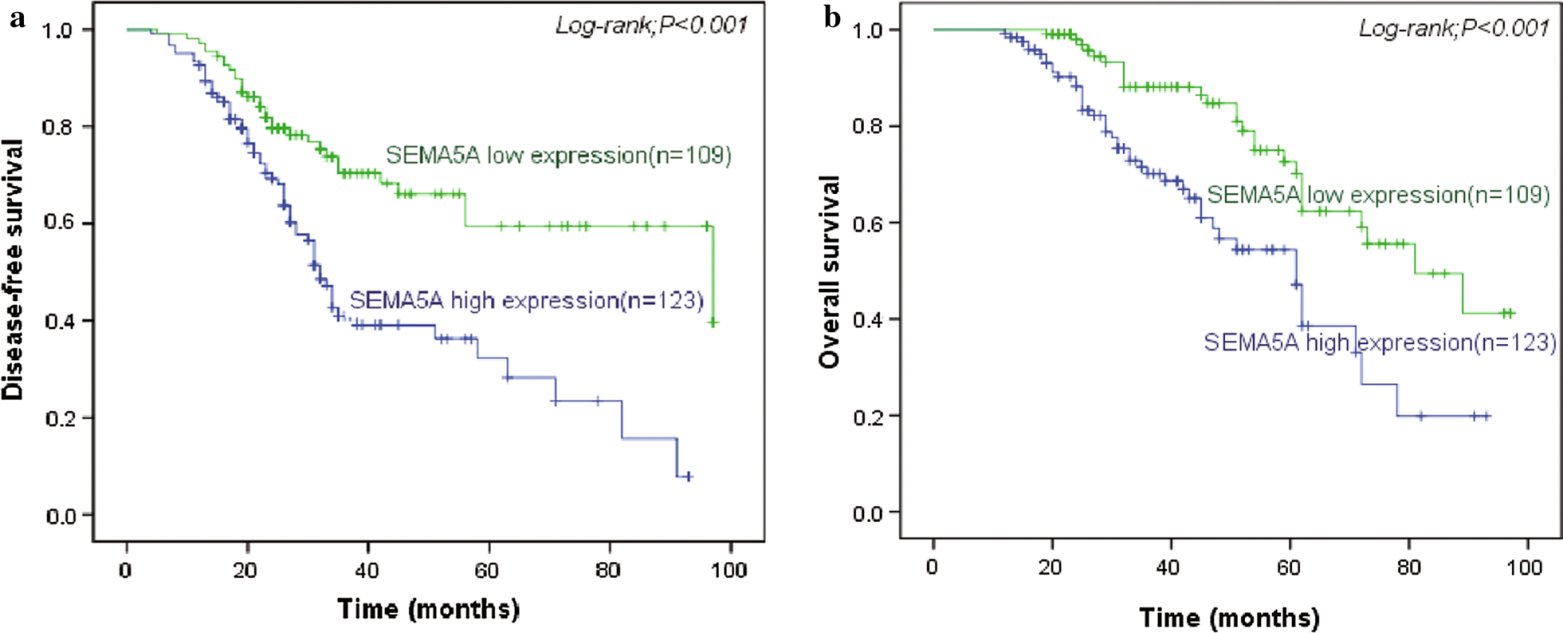
Disease-free and overall survival in cervical cancer patients according to SEMA5A expression. The log-rank test was used to analyze differences in disease-free and overall survival between the high and low SEMA5A expression groups. **a** Disease-free survival and **b** overall survival were significantly shorter for patients with high SEMA5A expression than for patients with low SEMA5A expression. *SEMA5A* semaphorin 5A

The mean follow-up interval was 40.6 months (range 12–97 months). During the follow-up interval, 95 patients (42.8%) developed recurrent disease. SEMA5A expression was significantly correlated with DFS. DFS was significantly (*P *< 0.001) lesser in patients with high tumor SEMA5A expression levels than for those with low tumor SEMA5A expression levels (Fig. 3a). High SEMA5A expression was also associated with a significantly shorter OS (*P *< 0.001; Fig. 3b). The median OS was 61.0 months in patients with high SEMA5A-expressing tumors (n = 123) and 81.0 months in patients with low SEMA5A-expressing tumors (n = 109). In the metastatic group (n = 121), median OS was 47.0 months in patients with high SEMA5A-expressing tumors (n = 80) and 73.0 months in patients with low SEMA5A-expressing tumors. The 5-year survival rates were 12.5 and 34.6% for the high and low SEMA5A expression groups, respectively. Therefore, high tumor SEMA5A expression level was a significant predictor of poor prognosis.

### Association of SEMA5A expression with clinicopathological parameters

The association between SEMA5A expression and various clinicopathological parameters is shown in Table 3. In the univariate analysis, tumor grade (*P* < 0.001), FIGO stage (*P* < 0.001), LNM (*P* < 0.001), presence of lymphatic invasion (*P* < 0.001), VEGF-C expression (*P* < 0.001), LMVD (*P* < 0.001), and SEMA5A expression (*P* = 0.004) were significant prognostic factors for OS. Multivariate analysis was performed to evaluate the independent prognostic role of SEMA5A after adjusting for other significant covariates. SEMA5A expression (*P* = 0.032), tumor grade (*P* < 0.001), FIGO stage (*P* < 0.001), LMN (*P* < 0.001), presence of lymphatic invasion (*P* < 0.001), VEGF-C expression (*P* < 0.001), and LMVD (*P* = 0.005) remained independent prognostic factors for OS in the multivariate analysis.

**Table 3:** Relationship between clinicopathological characteristics and SEMA5A expression in cervical cancer patients (n = 232)

Clinicopathological characteristics	SEMA5A expression		*P* value
	Low (n = 109)	High (n = 123)	
Age (years)			
≤ 45	47	60	0.388
> 45	62	63	
FIGO stage			
Ia	9	3	0.019^a^
Ib	44	35	
IIa	32	55	
IIb	24	30	
Tumor grade			
High	28	19	0.090
Intermediate	49	55	
Low	32	49	
Histological type			
Squamous carcinoma	76	80	0.262
Adenocarcinoma	29	32	
Adenosquamous carcinoma	4	11	
Lymphatic permeation			
No	96	94	0.021^a^
Yes	13	29	
VEGF-C expression			
High	19	101	< 0.001^a^
Low	90	22	
Lymphatic microvessel density			
High	23	92	< 0.001^a^
Low	86	31	
Lymph node metastasis			
No	68	43	< 0.001^a^
Yes	41	80	

*FIGO* Federation of Gynecology and Obstetrics, *SEMA5A* semaphorin 5A, *VEGF-C* vascular endothelial growth factor-C

^a^Chi square test *P* < 0.05

### SEMA5A induced VEGF-C expression by activating Met tyrosine kinase via plexin-B3

**Fig. 4:**
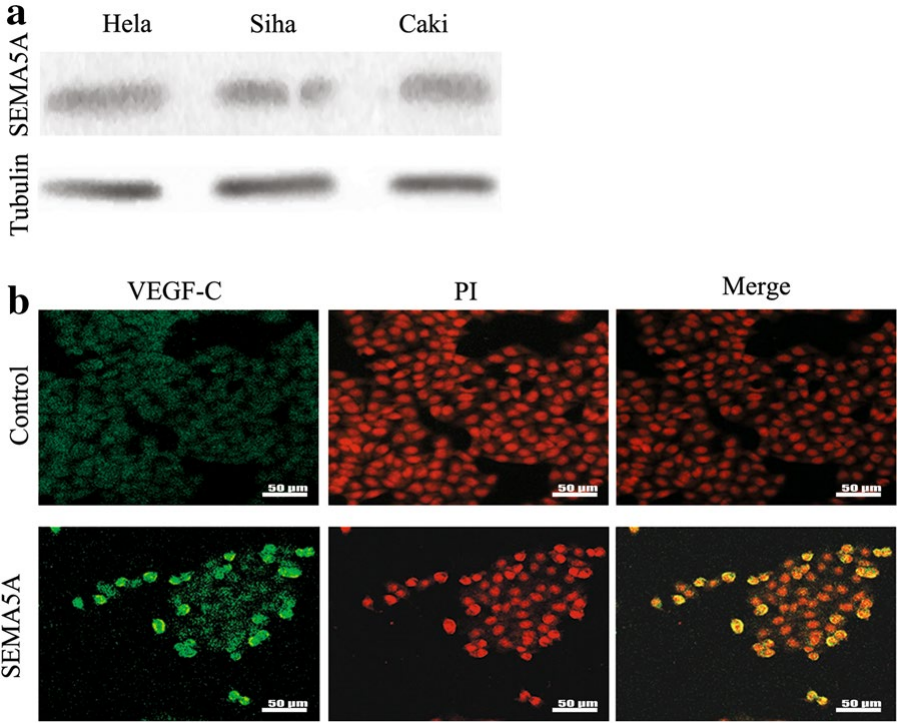
SEMA5A enhances VEGF-C expression in vitro in cervical cancer cells. **a** Western blot analysis demonstrated SEMA5A protein expression in Hela, Siha, and Caski cervical cancer cells. **b** Immunofluorescence staining showed increased VEGF-C expression in SEMA5A-stimulated (100 ng/mL) HeLa cervical cancer cells compared with the control. *SEMA5A* semaphorin 5A, *VEGF-C* vascular endothelial growth factor-C

**Fig. 5:**
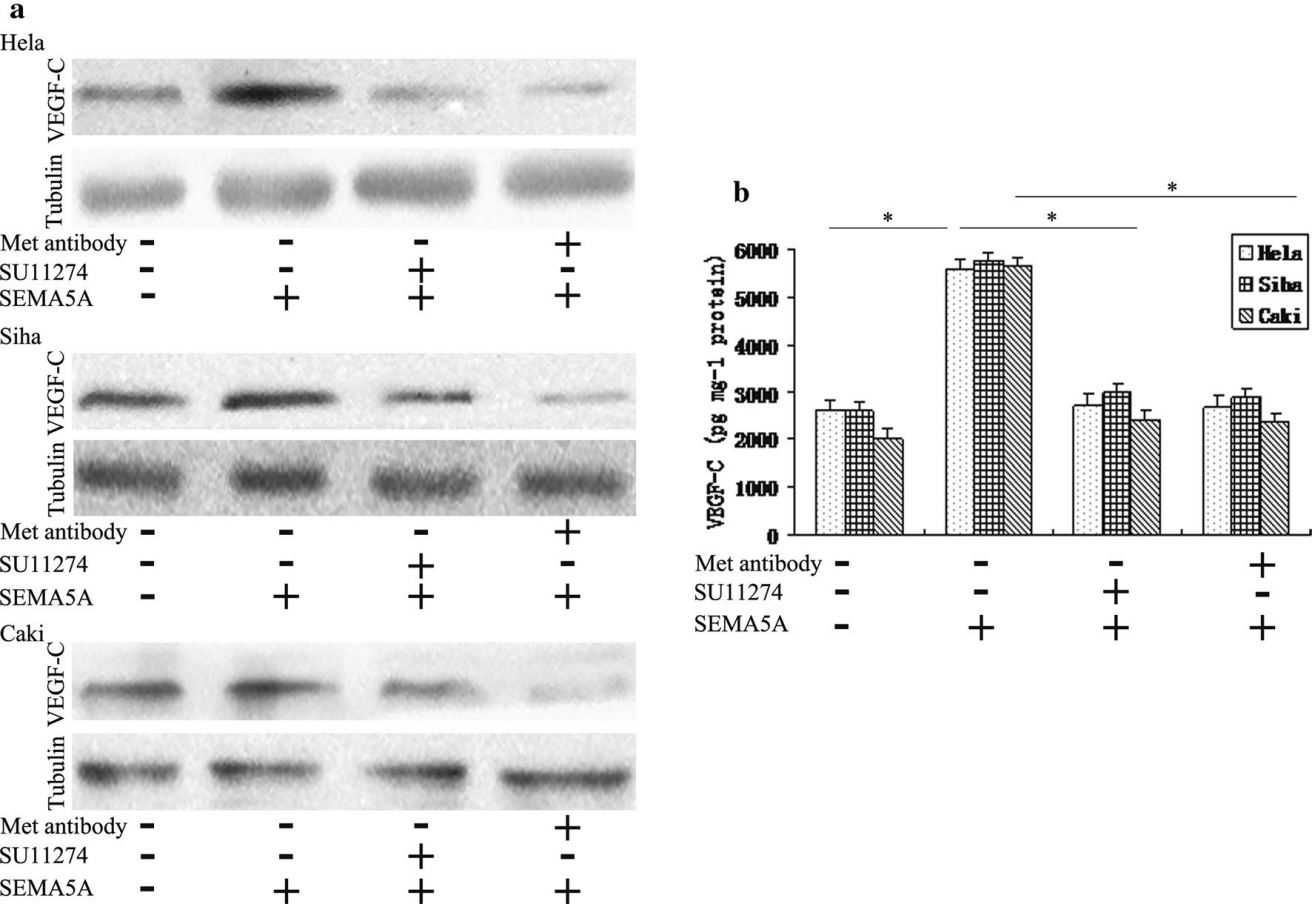
SEMA5A-induced VEGF-C expression is mediated through Met. **a** Western blot was used to determine VEGF-C protein expression in HeLa, Siha, and Caski cells treated with 100 ng/mL SEMA5A in the absence (−) or presence (+) of the Met inhibitor SU11274 (2.5 μM) or a Met neutralizing antibody (met antibody; 2 μg/mL). Tubulin was used as an internal loading control. Met inhibition suppressed the enhancement of VEGF-C expression by SEMA5A. **b** ELISA measurement of VEGF-C secretion was determined in HeLa, Siha, and Caski cells treated with 100 ng/mL SEMA5A in the absence (−) or presence (+) of SU11274 or Met antibody. VEGF-C ELISA results were consistent with the western blot results. Data shown are representative of at least three independent experiments. *P < 0.05 compared with basal level or SEMA5A alone (**b**). *SEMA5A* semaphorin 5A, *VEGF-C* vascular endothelial growth factor-C, *ELISA* enzyme-linked immunosorbent assay

We performed western blots for SEMA5A using protein lysates collected from in Hela, Siha, and Caski cervical cancer cells. We observed bands at ~ 135 and ~ 110 kDa (Additional file 2: Figure S2). They are membrane-bound SEMA5A and equivalent to the molecular weight of its extracellular domain, respectively [19]. The results shown in Fig. 4a revealed that SEMA5A expression was similar between Caski cells, which are derived from an intestinal metastasis of cervical cancer, and Hela and Siha cells, which derived from primary cervical cancer. However, our findings from cervical cancer patients suggested that SEMA5A was likely involved in the lymphangiogenesis and capacity of cervical cancer cells to metastasize to the lymph nodes. In view of the similar structural features between lymph and blood vessels, we speculated that SEMA5A may also affect the lymphatic system in vitro. In support of this, recombinant human SEMA5A has been shown to increase VEGF-C expression, which plays a key role in lymphangiogenesis [20]. Therefore, we determined the effect of SEMA5A on VEGF-C expression using fluorescent antibody staining and western blot. FITC-labeled VEGF-C localized to the cytoplasm and cytoplasmic surface of the plasma membrane in Hela cells. We found that SEMA5A enhanced VEGF-C expression compared with the control (Fig. 4b). Similar results were obtained in the western blot analysis of HeLa, Siha, and Caski cells (Fig. 5a).

**Fig. 6:**
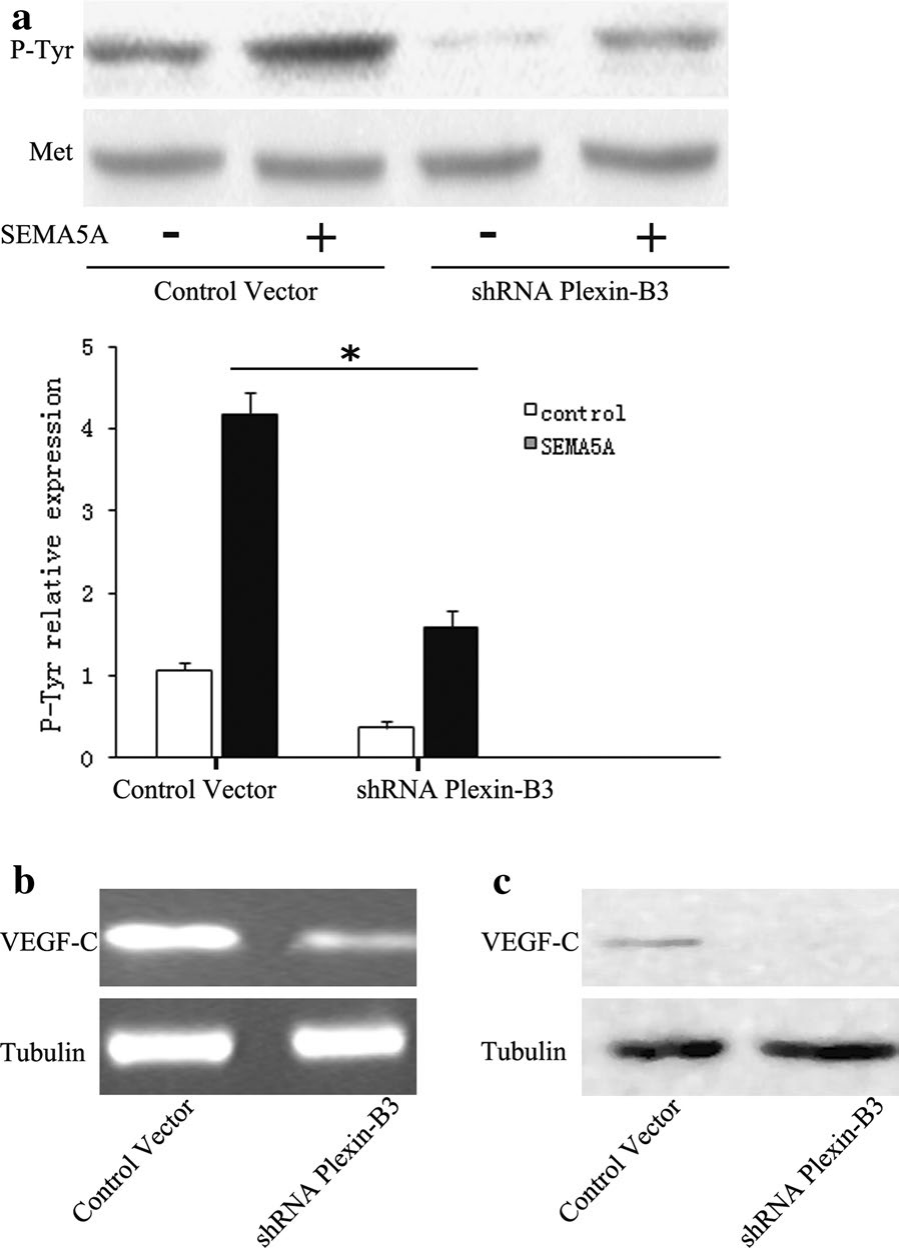
SEMA5A-induced Met phosphorylation and VEGF-C expression are dependent upon plexin-B3. **a** HeLa cells were transduced with lentiviruses expressing plexin-B3-specific shRNA or scrambled shRNA (Control Vector). Western blot analysis of Met phosphorylation was performed on shRNA-transfected cells stimulated with 100 ng/mL SEMA5A for 24 h. *Plexin*-*B3* knockdown reduced SEMA5A-induced Met phosphorylation. The bands were quantified and data are representative of at least three independent experiments. Columns, means of three experiments; bars, SD; *P < 0.05 vs. the respective controls. **b** RT-PCR and **c** western blot analysis of VEGF-C expression were performed on plexin-B3 shRNA and control shRNA-transfected HeLa cells. *Plexin*-*B* knockdown reduced VEGF-C mRNA and protein expression. RT-PCR, reverse transcription-polymerase chain reaction; SEMA5A, semaphorin 5A; shRNA, short hairpin RNA; VEGF-C, vascular endothelial growth factor-C

Recently, Met has been implicated in lymphangiogenesis through its association with VEGF-C [21]. Therefore, western blot analysis was used to determine whether SEMA5A induced VEGF-C expression through a Met-dependent signaling pathway. Inhibition of MET with the Met-specific inhibitor SU11274 or a Met neutralizing antibody decreased the SEMA5A-mediated enhancement of VEGF-C expression in Hela, Siha, and Caski cells (Fig. 5a). Qualitative measurement of VEGF-C levels by ELISA in the three cervical cancer cell lines was consistent with the western blot results (Fig. 5b). The known ligand for MET is HGF. Next, we examine whether Met receptor is involved in HGF-mediated VEGF-C production in Hela, Siha, and Caski cells. MET neutralizing antibody blocked HGF-stimulated VEGF-C mRNA and protein level. Furthermore, transfection with Met siRNA also inhibited HGF-increased VEGF-C production (Additional file 3: Figure S3A, B). In addition, SEMA5A treatment induced Met tyrosine phosphorylation (Fig. 6a).

SEMA5A has been found to interact with plexin-B3, a high-affinity SEMA5A-specific receptor [22, 23]. Moreover, plexin-B3 has also been proposed to interact with the receptor tyrosine kinase Met. Therefore, we tested whether SEMA5A-induced Met phosphorylation is dependent upon plexin-B3 using plexin-B3 RNA interference in HeLa cells. Western blot demonstrated *Plexin*-*B3* knockdown inhibited SEMA5A-induced Met phosphorylation. Quantifying the bands and the statistical analysis showed that SEMA5A signals through MET (Fig. 6a). Moreover, we tested whether plexin-B3 was involved in the induction of VEGF-C expression by SEMA5A. RT-PCR analysis showed that *plexin*-*B3* knockdown significantly decreased VEGF-C mRNA and protein expression compared with the control (Fig. 6b, c). Our results indicate that SEMA5A induces VEGF-C overexpression by activating Met signaling via plexin-B3.

### SEMA5A induces cervical cancer cell invasion by promoting MMP activity via a PI3K/AKT pathway

**Fig. 7:**
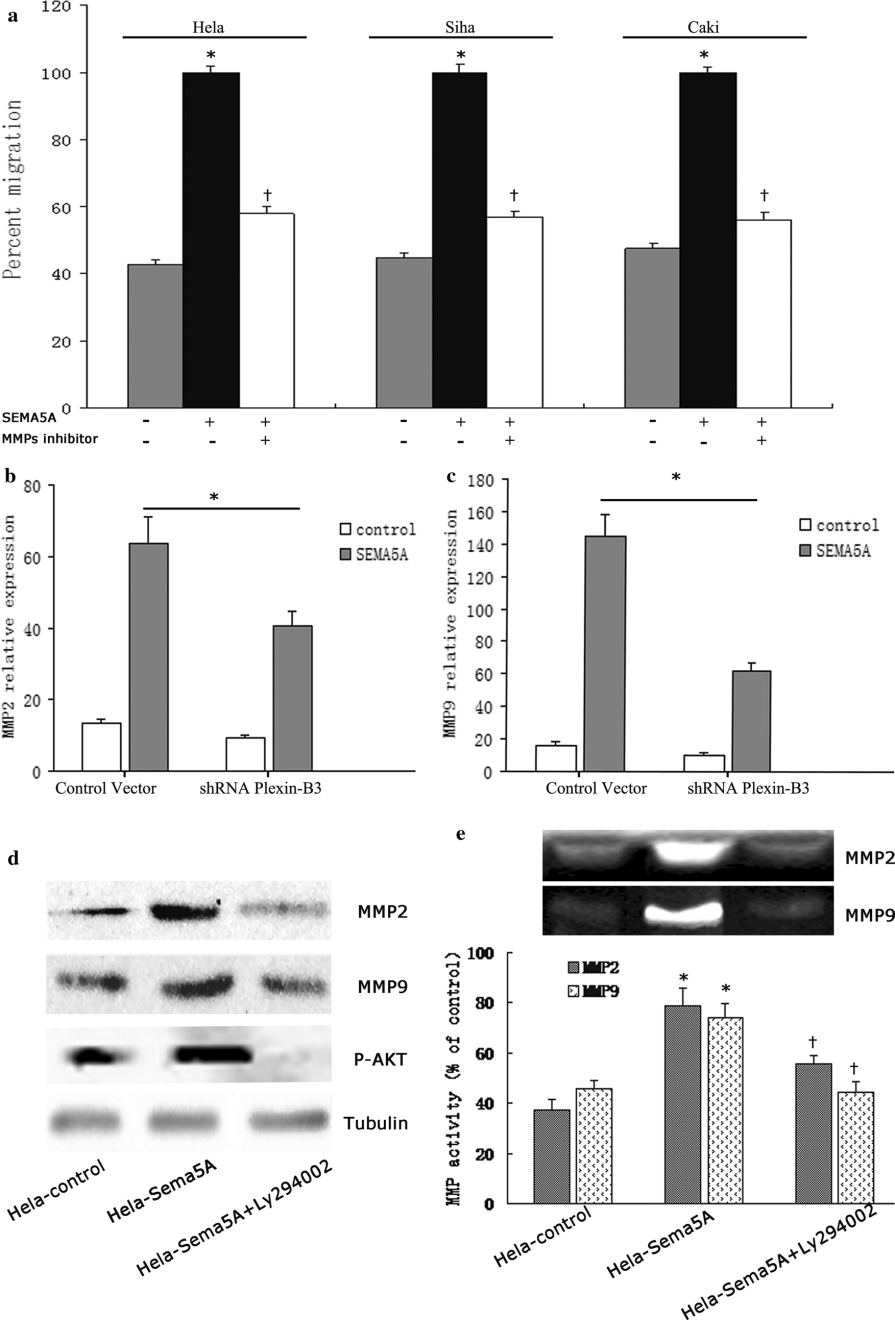
SEMA5A induces cervical cancer cell invasion by stimulating MMP activity via plexin-B3 and PI3K/AKT. **a** Matrigel invasion assays were performed on cervical carcinoma cell line Hela stimulated with or without SEMA5A (100 ng/mL) in the absence or presence of the MMP inhibitor GM6001 (1 μM). Data are shown as the percentage of invaded cells relative to the control. SEMA5A-induced invasion was dependent on MMP activity as evidenced by the decrease in SEMA5A-induced invasion with GM6001 treatment. *P < 0.05 vs. the respective controls. ^†^*P *< 0.05 vs. the respective SEMA5A-treated cells. *Plexin*-*B3* knockdown significantly decreased SEMA5A-induced (**b**) *MMP*-*2* and (**c**) *MMP*-*9* expression as assessed by real-time RT-PCR. **d** Western blot analysis showed a marked increase in the protein expression of MMP-2, MMP-9, and p-AKT in HeLa-Sema5A cells compared with HeLa-control cells. The PI3K inhibitor LY294002 reduced MMP-2, MMP-9, and p-AKT expression in HeLa-Sema5A cells. **e** Summary of MMP-2 and MMP-9 zymography data. Zymography data are representative of two independent experiments. The PI3K inhibitor LY294002 reversed the increase in MMP-2 and MMP-9 activities in HeLa-Sema5A cells. *P < 0.05 vs. the respective HeLa-control cells. ^†^*P *< 0.05 vs. the respective HeLa-Sema5A cells. Columns represent the mean of two triplicate experiments; bars represent the standard error. *MMP* matrix metalloproteinase, *p-AKT* phosphorylated AKT, *PI3K* phosphoinositide 3-kinase, *RT-PCR* reverse transcription-polymerase chain reaction, *SEMA5A* semaphorin 5A

To determine the proinvasive and prometastatic activities of SEMA5A, matrigel invasion assays were performed. SEMA5A treatment significantly increased the invasion of HeLa, Siha, and Caski cells relative to control cells. MMP-2 and MMP-9 can degrade type IV collagen, which contributes to tumor invasion and metastasis [24, 25]. Therefore, we determined whether SEMA5A-induced invasion involves MMP-2 and MMP-9. The MMP inhibitor GM6001 reduced the ability of SEMA5A to stimulate cervical cancer cell invasion (Fig. 7a). Next, we tested whether SEMA5A-induced MMP-2 and MMP-9 expression is dependent upon plexin-B3. *Plexin*-*B* knockdown strongly reduced SEMA5A-induced *MMP*-*2* and *MMP*-*9* in HeLa cells (Fig. 7b, c). Because the PI3K/AKT signaling pathway has been shown to increase tumor invasion and metastasis by regulating MMP-2 and MMP-9 [26, 27], we investigated the role of this pathway in SEMA5A-induced invasion. To do this, the protein expression and activity of MMP-2 and MMP-9 were determined in HeLa-Sema5A cells and HeLa-control cells treated with and without the PI3K inhibitor LY294002. MMP-2 and MMP-9 protein expression and activities were induced in HeLa-Sema5A cells but not in HeLa-control cells (Fig. 7d, e). Compared with the control, SEMA5A increased MMP-2 and MMP-9 activities by 52.7 and 38.2%, respectively. LY294002 reduced AKT phosphorylation to basal levels observed in control cells and nearly reversed the stimulatory action of SEMA5A on MMP-2 and MMP-9 expression and activities (Fig. 7d, e). Our findings indicate that the proinvasive activity of SEMA5A is associated with the induction of MMP-2 and MMP-9 in HeLa cervical cancer cells. Furthermore, the induction of MMP-2 and MMP-9 by SEMA5A is mediated by plexin-B3 and the PI3K/AKT pathway.

## Discussion

Most cancers spread predominantly by means of lymphatic vessels. Regional lymphatic metastasis is an important prognostic factor in many cancers, including cervical cancer [28]. A great deal of research has focused on two members of the VEGF family, VEGF-C and VEGF-D, which play a critical role in stimulating tumor lymphangiogenesis and lymphatic metastasis [29, 30]. Cao et al. [31] found that platelet-derived growth factor-BB is also involved in lymphangiogenesis and lymphatic metastasis. However, given the complexity of the metastatic process, it is likely that many factors are involved in its regulation. SEMA5A, an axon regulator, has been identified as a novel proangiogenic molecule. The similar structural and functional features of the blood and lymphatic systems raise the possibility that SEMA5A is also involved in lymphangiogenesis and lymphatic metastasis. In the present study, we attempted to elucidate the role of SEMA5A in cervical cancer by correlating its expression with clinicopathological features and prognosis. We provide evidence that SEMA5A promotes lymphangiogenesis and lymphatic invasion in vitro.

High SEMA5A expression level has been demonstrated in several cancers [9, 22, 32, 33]. However, the functional role of SEMA5A in tumor progression, lymphangiogenesis, and LNM in cervical cancer has not been reported. In present study, SEMA5A expression was elevated in stage IIb cervical cancer tissues compared with stage Ia, Ib, and IIa cervical cancer tissues. SEMA5A overexpression was also significantly associated with lymphangiogenesis, poor prognosis, and the metastatic potential of cervical cancer cells. We suggest that SEMA5A expression mainly plays a role in cervical cancer development at the primary site.

SEMA5A has been shown to promote angiogenesis [8]. In pancreatic cancer, it has been suggested that SEMA5A increases micrometastasis via VEGF-mediated increase in tumor angiogenesis [19]. In view of the similar structural features between blood and lymph vessels, we asked whether SEMA5A also influences the lymphatic system. We demonstrated that SEMA5A induces VEGF-C expression, one of the most potent direct-acting lymphangiogenic factors belonging to the VEGF family [34]. Sadanandam et al. [8] suggested that SEMA5A promotes angiogenesis by decreasing apoptosis through AKT activation and increasing endothelial cell migration through Met activation. A recent report provided indirect evidence for an association and possible regulatory link between Met and VEGF-C and lymphangiogenesis [21]. We found that Met is involved in SEMA5A-induced VEGF-C expression. Cumulative data suggests that certain semaphorins interact with their receptors to modulate cancer cell behavior and promote tumor development and angiogenesis by multiple mechanisms [35]. Plexin-B3, which belongs to the class B plexin subfamily, is a SEMA5A receptor. A growing body of evidence suggests that SEMA5A plays an important role in tumor progression through its interaction with plexin-B3 [33, 36, 37]. In view of these findings, we tested whether plexin-B3 is involved in the SEMA5A-mediated induction of VEGF-C expression by knocking down *plexin*-*B3* expression. Taken together, our results indicate that SEMA5A induces VEGF-C expression by activating Met via plexin-B3.

In present study, we demonstrated that SEMA5A contributes to cervical cancer cell invasion. MMPs degrade the extracellular matrix (ECM) and thus, facilitate tumor invasion and metastasis [38]. In particular, MMP-2 and MMP-9, which selectively degrade type IV collagen, have been shown to facilitate tumor invasion and metastasis in various cancers [39–43]. Studies have shown that the PI3K/AKT signaling pathway plays an important role in invasion and distant metastasis of cancer through MMP-2 and MMP-9 regulation [26, 27, 44, 45]. Consistent with these studies, we found that the PI3K/AKT pathway is involved in SEMA5A-mediated MMP-2 and MMP-9 expression and activities. Our data suggest that SEMA5A increases invasion by induction of MMP-2 and MMP-9 via the PI3K/AKT pathway.

LNM is common in cancer and increases the risk of recurrence. Our results substantiate that SEMA5A is associated with LNM in cervical cancer. We found that SEMA5A promotes lymphatic metastasis by three mechanisms. First, SEMA5A induces lymphangiogenesis and consequently increases the surface area of tumor cells in contact with lymphatic endothelial cells. Second, SEMA5A stimulates MMP-2 and MMP-9 to increase the invasive potential of cervical cancer cells. This involves plexin-B3 and the PI3K/AKT pathway. Finally, SEMA5A promotes lymphangiogenesis by activating Met-mediated VEGF-C expression via plexin B3.

Our results indicate that SEMA5A is prognostic indicator in cervical cancer. To our knowledge, this is the first study to evaluate patient outcomes in relation to SEMA5A expression level in cervical cancer. Lu et al. [5] reported that SEMA5A downregulation is associated with poor survival among nonsmoking women with non-small cell lung carcinoma. In contrast, our survival data provide compelling evidence that SEMA5A overexpression is associated with unfavorable outcome in cervical cancer. In addition, our data indicate that patients with high tumor SEMA5A expression levels are significantly more likely to develop metastases than those with low tumor SEMA5A expression levels. Semaphorins have been reported to have dual roles in cancer. Semaphorins act as putative tumor suppressors and antiangiogenic factors in certain cancers and as mediators of tumor angiogenesis, invasion, and metastasis in others [8, 22, 32, 46]. Previous studies have indicated that the effect of SEMA5A on clinical outcome is dependent on its functional role [47, 48]. Semaphorin receptor complexes and their downstream signaling pathways vary according to cancer type [49]. Therefore, differences in SEMA5A function may be due to differences in tumor biology.

Although we found that high-expression of SEMA5A may accelerate LNM and progression of cervical cancer, the limitations of the study should be noted. Further studies are needed to fully understand the underlying molecular pathways of SEMA5A in cervical cancer. An understanding of these mechanistic pathways is required for the potential clinical application of SEMA5A as a molecular marker of tumor metastasis and potential prognostic marker.

## Conclusions

This study demonstrated for the first time a link between SEMA5A overexpression and lymphangiogenesis, LNM, and reduced survival in cervical cancer patients. Our study also provided evidence that SEMA5A promoted lymphangiogenesis and invasion through different molecular pathways. These data afford a comprehensive view of a novel function for SEMA5A, which could be a potential indicator of cervical cancer. Collectively, our findings may be important in the identification of high-risk cervical cancer patients and thus, improve their clinical management. Furthermore, our findings have important implications for the development of molecular targeted therapies to treat not only aggressive cervical cancers but to prevent or delay disease recurrence.

## Additional files

**Additional file 1: Figure S1.** Positive and negative control tissues demonstrated the specificity of SEMA5A antibody. The section using PBS instead of the SEMA5A antibody was shown as a negative control (a). The section of nonsmall cell lung carcinoma was shown as a positive control (b). ×200 magnification. SEMA5A, semaphorin 5A.

**Additional file 2: Figure S2.** A representative western blot analysis showed 135 and 110 kDa bands of SEMA5A in protein lysates of Hela, Siha, and Caski cervical cancer cells.

**Additional file 3: Figure S3.** MET regulates VEGF-C expression through HGF. (a, b) HeLa, Siha, and Caski cells were pretreated with the Met neutralizing antibody (Met antibody; 2 μg/mL) or transfected with Met siRNA for 24 h followed by treatment with HGF for 24 h, the VEGF-C expression was examined by RT-PCR and ELISA. Data shown are representative of at least three independent experiments. ^†^*P *< 0.05 compared with control; *P < 0.05 compared with HGF-treated group (a, b). HGF, hepatcyte growth factor; RT-PCR, Quantitative real-time reverse transcription-polymerase chain reaction.
