# Supplementary figures and images for: The association of semaphorin 5A with lymph node metastasis and adverse prognosis in cervical cancer
Source: Cancer Cell Int. 2018 Jun 22;18:87. doi: 10.1186/s12935-018-0584-1 (PMC6013961; doi:10.1186/s12935-018-0584-1)

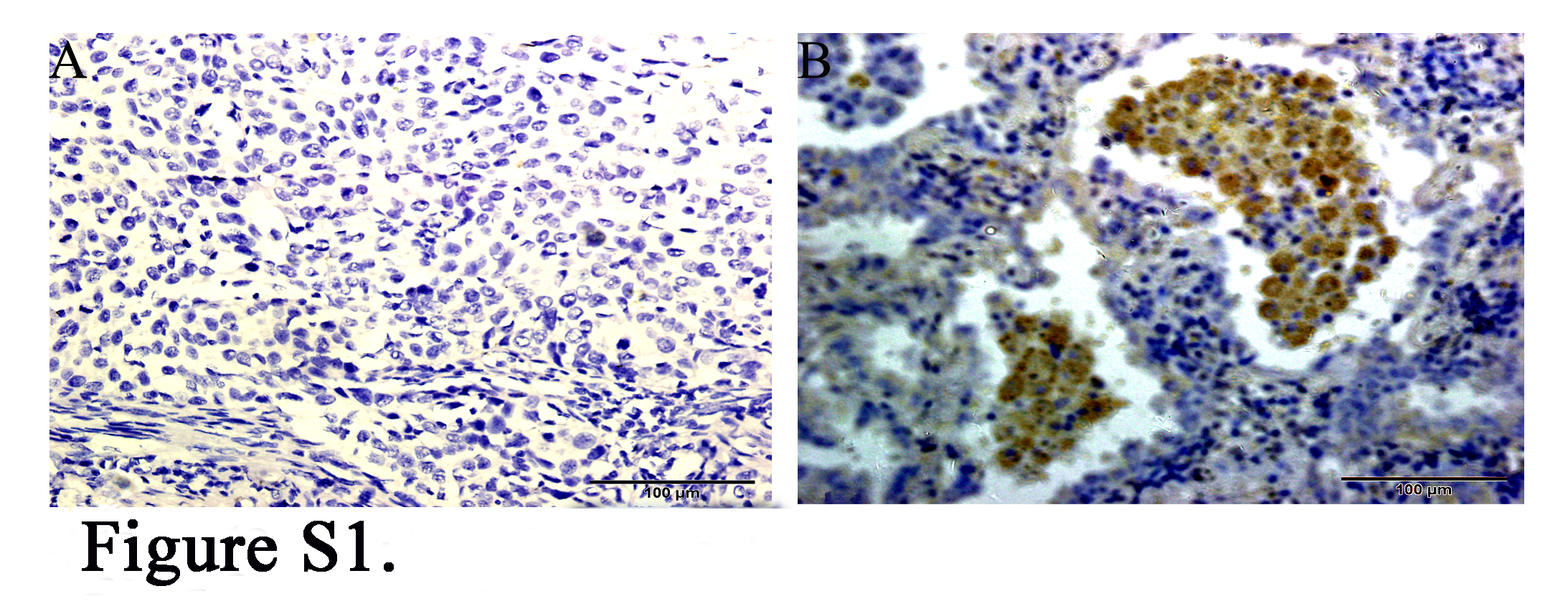

Supplement: Supplementary file 1 — Additional file 1: Figure S1. Positive and negative control tissues demonstrated the specificity of SEMA5A antibody. The section using PBS instead of the SEMA5A antibody was shown as a negative control (a). The section of nonsmall cell lung carcinoma was shown as a positive control (b). ×200 magnification. SEMA5A, semaphorin 5A. [file 12935_2018_584_MOESM1_ESM.tif]

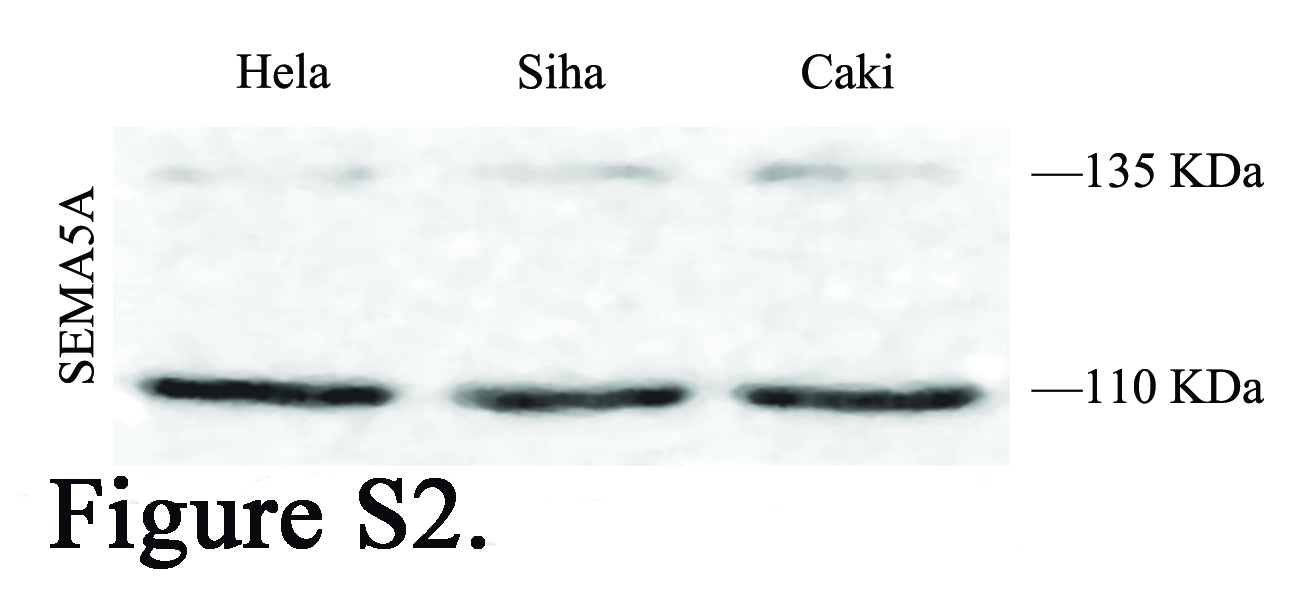

Supplement: Supplementary file 2 — Additional file 2: Figure S2. A representative western blot analysis showed 135 and 110 kDa bands of SEMA5A in protein lysates of Hela, Siha, and Caski cervical cancer cells. [file 12935_2018_584_MOESM2_ESM.tif]

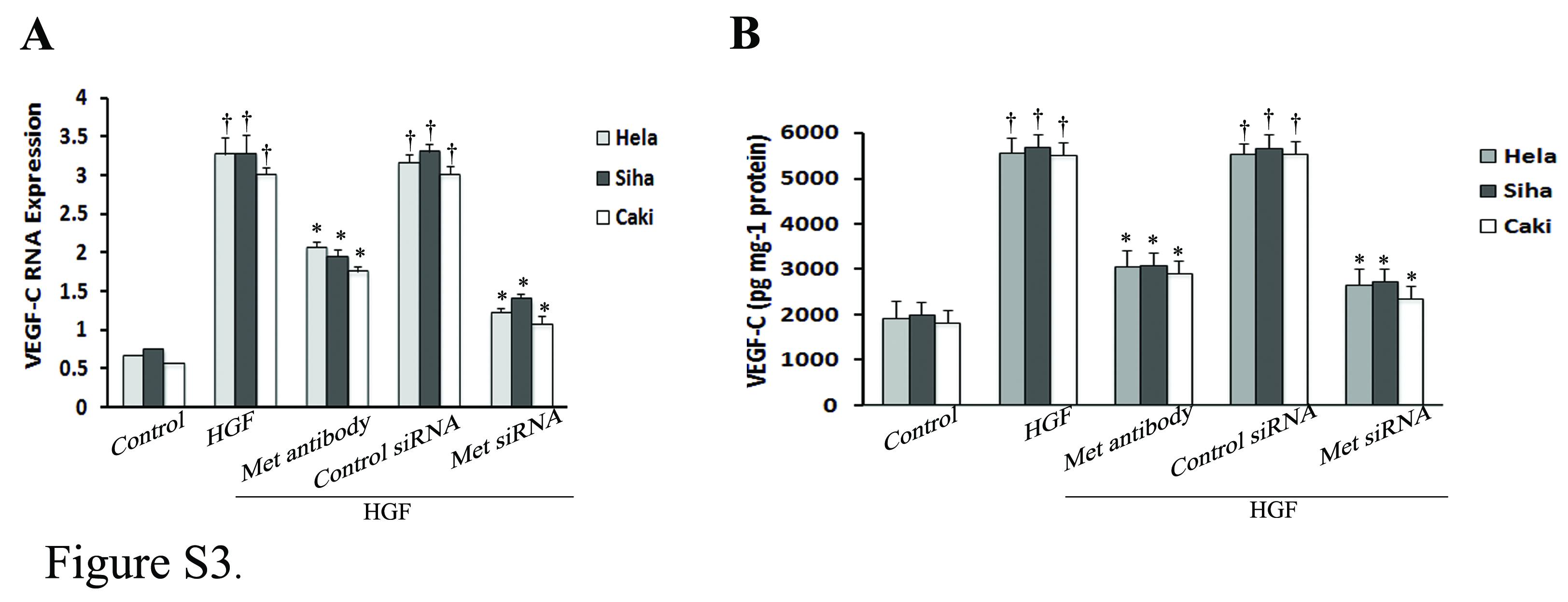

Supplement: Supplementary file 3 — Additional file 3: Figure S3. MET regulates VEGF-C expression through HGF. (a, b) HeLa, Siha, and Caski cells were pretreated with the Met neutralizing antibody (Met antibody; 2 μg/mL) or transfected with Met siRNA for 24 h followed by treatment with HGF for 24 h, the VEGF-C expression was examined by RT-PCR and ELISA. Data shown are representative of at least three independent experiments. †P < 0.05 compared with control; *P < 0.05 compared with HGF-treated group (a, b). HGF, hepatcyte growth factor; RT-PCR, Quantitative real-time reverse transcription-polymerase chain reaction. [file 12935_2018_584_MOESM3_ESM.tif]
